# BCG vaccination potentiates oxidative phosphorylation in neonatal myeloid‐derived suppressor cells

**DOI:** 10.1002/2211-5463.70333

**Published:** 2026-08-23

**Authors:** Yingying Chen, Hui Li

**Affiliations:** ^1^ Department of Clinical Laboratory, State Key Laboratory of Respiratory Disease, National Center for Respiratory Medicine, National Clinical Research Center for Respiratory Disease The First Affiliated Hospital of Guangzhou Medical University China; ^2^ Tianjin Institute of Immunology, Key Laboratory of Immune Microenvironment and Disease of the Ministry of Education, Department of Immunology, School of Basic Medical Sciences Tianjin Medical University China

**Keywords:** Bacillus Calmette‐Guérin, immunometabolism, immunosuppressive function, myeloid‐derived suppressor cells, oxidative phosphorylation

## Abstract

Bacillus Calmette–Guérin (BCG) vaccination has well‐documented off‐target effects in early life, but the underlying molecular mechanisms remain incompletely understood. This study investigated whether BCG reprograms neonatal myeloid‐derived suppressor cells (MDSCs) through oxidative phosphorylation (OXPHOS). After neonatal mice received a subcutaneous injection of BCG or PBS, the effects of BCG exposure on splenic monocytic (M‐) and polymorphonuclear (PMN‐) MDSCs were assessed. BCG vaccination significantly increased the expression of OXPHOS‐related genes (*Ndufab1, Sdhd, Uqcrfs1, Cox4i*) and mitochondrial oxidative activity in both MDSC subsets. Seahorse analysis revealed a higher oxygen consumption rate in BCG‐exposed PMN‐MDSCs. Functional assays showed that BCG impaired MDSC‐mediated T‐cell suppression, with oligomycin treatment partially able to restore this suppressive capacity. Together, these findings indicate that BCG‐induced OXPHOS potentiation contributes to the loss of MDSC immunosuppressive function in neonatal mice, providing a molecular mechanism for the non‐specific immunomodulatory effects of BCG vaccination early in life.

## Graphical abstract

BCG vaccination enhances oxidative phosphorylation in neonatal MDSCs, impairing their immunosuppressive function. It upregulates electron transport chain genes and mitochondrial activity, increasing ATP and oxygen consumption. Pharmacological OXPHOS inhibition partially restores suppressive capacity, confirming causality. This identifies a metabolic mechanism underlying BCG's heterologous effects in early life.

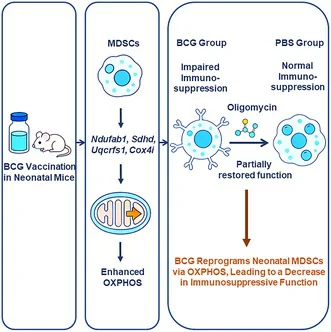

**Abbreviations**
AA: antimycin A
BCG: Bacillus Calmette‐Guérin
CCA: canonical correlation analysis
cDNA: complementary DNA
CFSE: carboxyfluorescein succinimidyl ester
CFU: colony‐forming units
DMSO: dimethyl sulfoxide
EDTA: ethylenediaminetetraacetic acid
FACS: fluorescence‐activated cell sorting
FBS: fetal bovine serum
FCCP: carbonyl cyanide‐4‐(trifluoromethoxy)phenylhydrazone
FDR: false discovery rate
GEMs: gel beads‐in‐emulsion
GO: Gene Ontology
GSEA: gene set enrichment analysis
KEGG: Kyoto Encyclopedia of Genes and Genomes
MDSCs: myeloid‐derived suppressor cells
MFI: mean fluorescence intensity
M‐MDSCs: monocytic myeloid‐derived suppressor cells
MSigDB: Molecular Signatures Database
OCR: oxygen consumption rate
OVA: ovalbumin
OXPHOS: oxidative phosphorylation
PBS: phosphate‐buffered saline
PCA: principal component analysis
PMN‐MDSCs: polymorphonuclear myeloid‐derived suppressor cells
qRT‐PCR: quantitative real‐time polymerase chain reaction
RNA‐seq: RNA sequencing
RTN: rotenone
scRNA‐seq: single‐cell RNA sequencing
SEM: standard error of the mean
TB: tuberculosis
UMAP: uniform manifold approximation and projection
UMIs: unique molecular identifiers

Bacillus Calmette‐Guérin (BCG), a live‐attenuated vaccine originally developed to prevent tuberculosis, is known to confer substantial non‐specific or heterologous benefits in addition to its target protection [1, 2]. These off‐target effects are linked to a marked reduction in all‐cause infant mortality, largely by enhancing resistance to a range of unrelated pathogens [3, 4]. The phenomenon is widely attributed to “trained immunity,” a persistent functional state of the innate immune system driven by long‐term metabolic and epigenetic reprogramming of monocytes and macrophages [5, 6]. Enhanced glycolysis via the mTOR‐HIF1α axis is a key metabolic feature underlying this hyper‐responsive phenotype [7, 8].

The neonatal period is a critical window for immune development, during which the immune system is inherently biased toward tolerance, helping to establish commensal microbiota and prevent excessive inflammation [9]. In extremely premature infants (born before 30 weeks of gestation), circulating immune cells follow distinct developmental trajectories compared to full‐term infants [10]. Myeloid‐derived suppressor cells (MDSCs), a heterogeneous group of immature myeloid cells that includes monocytic (M‐MDSCs) and polymorphonuclear (PMN‐MDSCs) subsets, are markedly expanded during this period [11, 12]. They help maintain immune homeostasis early in life by restraining overactive T‐cell responses [13]. Disruption of neonatal MDSC homeostasis has been implicated in inflammatory diseases such as necrotizing enterocolitis [14, 15].

We previously reported that BCG vaccination impairs the immunosuppressive function of both MDSC subsets in neonatal mice through an mTOR‐HIF1α‐dependent upregulation of glycolysis [16, 17]. Glycolysis is a well‐established pathway in immune activation, but the role of oxidative phosphorylation (OXPHOS), the main energy pathway in quiescent and alternatively activated cells, remains poorly understood in neonatal MDSCs. Given the growing complexity of immunometabolism, we hypothesized that BCG vaccination might induce broader metabolic rewiring in these cells beyond glycolysis.

To test this hypothesis, we employed an integrated approach that combined re‐analysis of single‐cell and bulk RNA‐seq data with metabolic profiling and functional assays to characterize BCG‐induced metabolic reprogramming in neonatal MDSCs. We show that BCG vaccination enhances OXPHOS in both M‐MDSC and PMN‐MDSC subsets by upregulating components of the electron transport chain. Furthermore, using the pharmacological OXPHOS inhibitor oligomycin, we establish a causal link between BCG‐driven OXPHOS augmentation and the loss of MDSC immunosuppressive capacity. A recent study demonstrated that kinetic modeling of cellular metabolic responses can improve interpretability of *in vitro* glycolysis assays and provide deeper insight into metabolic states [18]. Our work similarly benefits from a quantitative, integrated analysis of OXPHOS activity in the context of post‐vaccination immune cell reprogramming.

## Materials and methods

### Mice

All animal procedures were approved by the Institutional Animal Care and Use Committee of Tianjin Medical University (TMUaMEC2024057). Wild‐type C57BL/6 mice came from the Laboratory Animal Center of Tianjin Medical University (Tianjin, China). For timed pregnancies, paired mating was set up twice a week. Pregnant females were housed individually and checked twice daily for delivery; only full‐term pups were used. Each experimental group had a roughly equal sex distribution, and littermates from the same cage served as controls. All pups stayed with their dams under specific pathogen‐free conditions throughout the study. Mice were housed in individually ventilated cages with sterilized bedding, under a 12‐h light/dark cycle, with free access to food and water, at 22 ± 2 °C and 50 ± 10% relative humidity. We took care to minimize animal suffering by gentle handling and, when needed, used isoflurane anesthesia prior to rapid decapitation with sharp scissors for euthanasia, which is approved by AVMA for neonatal rodents. Immediately after euthanasia, cells were harvested for downstream assays. The experimental design and reporting followed the ARRIVE 2.0 guidelines; the essential 10 items are incorporated in the methods and results sections. The methodology used followed the Animal Experiment Ethics Requirements of Tianjin Medical University.

### Murine BCG vaccination

Lyophilized BCG (Chengdu Institute of Biological Products, Chengdu, China) was stored at 4 °C and reconstituted according to the manufacturer's instructions immediately prior to injection. As previously described [19, 20], neonatal mice (3‐ to 4‐day‐old) received a single subcutaneous injection of 5 × 10^5^ colony‐forming units (CFU) of BCG to meet the specific requirements of each experiment. For phenotypic detection of M‐MDSCs or PMN‐MDSCs, neonatal mice were injected with either BCG or PBS, with *n* = 4 mice per group.

### Flow cytometric analysis and sorting

As previously described, single‐cell suspensions were initially stained with antibodies against surface antigens for 30 min at 4 °C using cold fluorescence‐activated cell sorting (FACS) buffer (1× PBS supplemented with 1% FBS and 2 mm EDTA). Viable cells were distinguished using a LIVE/DEAD viability dye (Thermo Fisher Scientific, Waltham, MA, USA). Flow cytometric analysis was conducted on a CytoFLEX flow cytometer (Beckman Coulter, Brea, CA, USA), and sorting was performed on a MoFlo Astrios EQs cell sorter (Beckman Coulter). Data analysis was carried out with flowjo v10 software (TreeStar, Ashland, OR, USA). The following gating strategies were applied for cell identification: CD11b^+^Ly6C^hi^Ly6G^−^ for mouse M‐MDSCs, CD11b^+^Ly6G^+^Ly6C^lo^ for PMN‐MDSCs, and CD11b^+^Gr1^+^ for MDSCs. The following fluorescently labeled anti‐mouse antibodies (BioLegend, San Diego, CA, USA) were used in this study: PE Anti‐mouse CD3 (clone 17A2), APC/Cyanine7 Anti‐mouse CD4 (clone GK1.5), APC Anti‐mouse CD4 (clone GK1.5), APC/Cyanine7 Anti‐mouse CD8a (clone 53–6.7), APC Anti‐mouse CD8a (clone 53–6.7), FITC Anti‐mouse CD11b (clone M1/70), Brilliant Violet 421 Anti‐mouse CD11b (clone M1/70), PE Anti‐mouse Ly6G (clone 1A8), PE/Cyanine7 Anti‐mouse Ly6G (clone 1A8), APC Anti‐mouse Ly6C (clone HK1.4), Percp‐cy5.5 Anti‐mouse Ly6C (clone HK1.4), APC Anti‐mouse CD19 (clone 1D3/CD19).

### Assessment of MDSC immunosuppressive function

Neonatal mice (postnatal days 3–4) were subcutaneously vaccinated with BCG (5 × 10^5^ CFU). M‐MDSC suppression assay: M‐MDSCs were isolated from the spleens of neonatal mice 3 days post‐vaccination. CD3^+^ T cells were isolated from the spleens of adult mice, labeled with CFSE (2 μm; Thermo Fisher Scientific), and stimulated with plate‐bound anti‐CD3 (5 μg·mL^−1^; Invitrogen, Carlsbad, CA, USA) and soluble anti‐CD28 (1 μg·mL^−1^; eBioscience, San Diego, CA, USA). These activated T cells were then cultured alone or co‐cultured with M‐MDSCs at a ratio of 8 : 1 (T cells to M‐MDSCs) for 3 days. T cell proliferation was assessed by measuring CFSE dilution using a CytoFLEX flow cytometer (Beckman Coulter) (*n* = 3–4 mice per group). PMN‐MDSC suppression assay: PMN‐MDSCs were isolated from the spleens of neonatal mice 3 days post‐vaccination. Spleen cells from OT‐I mice were mixed with CD8^+^ T cells from wild‐type mice at a 1 : 4 ratio. The cell mixture was labeled with CFSE (2 μm) and then co‐cultured with PMN‐MDSCs at a 2 : 1 ratio (mixed cells to PMN‐MDSCs) in the presence of the cognate OVA_2₅₇‐2₆₄_ peptide (SIINFEKL, 0.5 ng·mL^−1^; Sigma‐Aldrich, St. Louis, MO, USA). After 60 h of incubation, T cell proliferation was determined based on CFSE dilution by flow cytometry on a CytoFLEX instrument (*n* = 3–4 mice per group). For *in vitro* functional assay of oligomycin, splenic M‐MDSCs and PMN‐MDSCs were subsequently isolated from these vaccinated mice and subjected to *in vitro* culture for 24 h in the presence of either DMSO (vehicle control) or 1 μm oligomycin (Sigma‐Aldrich) prior to functional assays (*n* = 3–4 mice per group).

### Quantitative real‐time PCR (qRT‐PCR)

As previously reported [21], total RNA was extracted from samples using TRIzol reagent (Thermo Fisher Scientific), following the manufacturer's protocol. Complementary DNA (cDNA) was then synthesized from the extracted RNA using the StarScript III First‐Strand cDNA Synthesis Kit (Genstar, Beijing, China). Quantitative PCR was performed with SYBR Green qPCR Master Mix (Genstar) on a real‐time PCR system. Gene expression levels were quantified using the comparative *C*_t_ ($2^{-\Delta \Delta C_{t}}$) method, with β‐actin serving as the endogenous control for normalization. All primer sequences used in this study are provided in Table 1. For each gene, *n* = 3–6 mice per group were analyzed.

**Table 1:** Primer sequences used in qRT‐PCR. *Atp5a*, ATP synthase, H+ transporting, mitochondrial F1 complex, alpha subunit 1; *Cox4i*, Cytochrome c oxidase subunit IV isoform 1; *mt‐Co1*, cytochrome c oxidase I, mitochondrial; *mt‐Co2*, cytochrome c oxidase II, mitochondrial; *Ndufab1*, NADH dehydrogenase (ubiquinone) 1, alpha/beta subcomplex 1; *Ndufs3*, NADH dehydrogenase (ubiquinone) Fe‐S protein 3; *Sdhb*, Succinate dehydrogenase complex, subunit B; *Sdhd*, Succinate dehydrogenase complex, subunit D, integral membrane protein; *Uqcrfs1*, Ubiquinol‐cytochrome c reductase, rieske iron–sulfur polypeptide 1.

Primers	Sequences
*β‐Actin*	Forward: 5′‐GACGGCCAGGTCATCACTATTG‐3′ Reverse: 5′‐AGGAAGGCTGGAAAAGAGCC‐3′
*Ndufab1*	Forward: 5′‐ATGGCGTCTCGTGTCCTCT‐3′ Reverse: 5′‐GCGGCACAAATGTGTGACT‐3′
*Ndufs3*	Forward: 5′‐TGGCAGCACGTAAGAAGGG‐3′ Reverse: 5′‐CTTGGGTAAGATTTCAGCCACAT‐3′
*Sdhb*	Forward: 5′‐AATTTGCCATTTACCGATGGGA‐3′ Reverse: 5′‐AGCATCCAACACCATAGGTCC‐3′
*Sdhd*	Forward: 5′‐TGGTCAGACCCGCTTATGTG‐3′ Reverse: 5′‐GGTCCAGTGGAGAGATGCAG‐3′
*Uqcrfs1*	Forward: 5′‐GAGCCACCTGTTCTGGATGTG‐3′ Reverse: 5′‐GCACGACGATAGTCAGAGAAGTC‐3′
*Cox4i*	Forward: 5′‐ATTGGCAAGAGAGCCATTTCTAC‐3′ Reverse: 5′‐CACGCCGATCAGCGTAAGT‐3′
*Atp5a*	Forward: 5′‐TCTCCATGCCTCTAACACTCG‐3′ Reverse: 5′‐CCAGGTCAACAGACGTGTCAG‐3′
*mt‐Co1*	Forward: 5′‐TCCAACTCATCCCTTGACATC‐3′ Reverse: 5′‐TCCTGCTATGATAGCAAACACT‐3′
*mt‐Co2*	Forward: 5′‐CTAATTAGCTCCTTAGTCCTC‐3′ Reverse: 5′‐TTCGTAGCTTCAGTATCATTG‐3′

### Single‐cell RNA sequencing (scRNA‐seq) and re‐analysis

We re‐analyzed our previously published datasets of scRNA‐seq (GSE234153) from splenic CD3^−^CD19^−^CD11b^+^ myeloid cells of BCG‐vaccinated and PBS‐treated neonatal mice. As described [16, 17], neonatal mice (3–4 days old) received a subcutaneous injection of either BCG (5 × 10^5^ CFU) or PBS (*n* = 1 mouse per group, with cells pooled from each group). Three days post‐vaccination, CD3^−^CD19^−^CD11b^+^ splenic cells were isolated by FACS and processed using the 10× Genomics Chromium platform (10× Genomics, Pleasanton, CA, USA). Gel beads‐in‐emulsion (GEMs) were generated, followed by cell lysis and barcoding of RNA with unique molecular identifiers (UMIs). Libraries were constructed with the Chromium Single Cell 3′ Reagent Kit and sequenced on the Illumina NovaSeq platform (Illumina, San Diego, CA, USA). Raw sequencing data were aligned to the mouse reference genome (mm10) using Cell Ranger (v6.1.2) [22]. The gene‐cell matrices were imported into Seurat (v4.0.4) [23] in R (v4.1.0) [24]. Quality control excluded cells with < 500 or > 7500 detected genes or > 5% mitochondrial reads, retaining 21 269 high‐quality transcriptomes. The top 2000 variable genes were integrated using canonical correlation analysis (CCA) [25]. Dimensionality reduction was performed via PCA (42 principal components), and cell clustering was carried out at a resolution of 0.4. Cell types were annotated using established marker genes. Differential expression analysis was conducted with FindAllMarkers, and pathway scoring was performed using AddModuleScore. Sub‐clustering of PMN‐MDSCs used 20 principal components and a resolution of 0.1, visualized by UMAP. All plots were generated using Seurat visualization functions. Gene set enrichment analysis (GSEA) was conducted to evaluate the enrichment of biological pathways and signature gene sets in PMN‐MDSCs from the BCG and PBS groups. The analysis was performed with the clusterprofiler package (version 4.4.2). Gene sets used in the GSEA were sourced from the Hallmark collections of the Molecular Signatures Database (msigdb, version 7.5.1) [26, 27, 28] with a total of 50 signature datasets included in the assessment.

### Bulk RNA sequencing (RNA‐seq) and re‐analysis

We re‐analyzed our previously datasets of bulk RNA‐seq data for splenic M‐MDSCs (GSE330508) and PMN‐MDSCs (GSE234153) from BCG‐vaccinated and PBS‐treated neonates [16, 17]. Splenic M‐MDSCs (*n* = 2 mice per group) and PMN‐MDSCs (*n* = 2 mice per group) were isolated by FACS from neonatal mice 3 days after BCG vaccination (5 × 10^5^ CFU). Total RNA was extracted using TRIzol reagent, and cDNA libraries were prepared with M‐MuLV Reverse Transcriptase and Phusion High‐Fidelity DNA Polymerase. Sequencing was performed on the Illumina NovaSeq platform in 2 × 150 bp paired‐end mode. Raw reads were aligned to the mm10 genome using star (v2.7.9a) [29], and gene expression was quantified with rsem (v1.2.28) [30]. Differential expression analysis was conducted with deseq2 (v1.36.0) [31], with multiple testing correction via the Benjamini–Hochberg method (FDR < 0.05). Genes with |log_2_(fold change)| > 1 and adjusted *P* < 0.05 were considered differentially expressed. KEGG pathway enrichment analysis was performed using clusterprofiler (v4.4.2) [32].

### Seahorse analysis

Neonatal mice (3–4 days old) were subcutaneously vaccinated with BCG (5 × 10^5^ CFU). PMN‐MDSCs were isolated from the spleens of these mice 3 days post‐vaccination; the oxygen consumption rate (OCR) was measured in real‐time using a Seahorse XFe96 Flux Analyzer (Agilent Technologies, Santa Clara, CA, USA). Prior to the assay, Seahorse microplates were coated with 22.4 μg·mL^−1^ Cell‐Tak (Corning, Corning, NY, USA) in 200 mm sodium bicarbonate solution. Approximately 1 × 10^5^ freshly isolated PMN‐MDSCs were seeded per well in OCR assay medium and treated sequentially with oligomycin (5 μm), FCCP (1 μm), and a mix of rotenone (RTN, 0.75 μm) and antimycin A (AA, 1 μm) using the Seahorse XF Cell Mito Stress Test Kit (Agilent Technologies), according to the manufacturer's instructions. For this assay, *n* = 4 mice per group were used.

### Analysis of MitoTracker by flow cytometry

Freshly isolated cells were assessed by staining with 50 nm MitoTracker Red FM (Thermo Fisher Scientific) for 30 min at 37 °C under 5% CO_2_. Following incubation, cells were washed twice with cold PBS and subsequently stained with surface antibodies for flow cytometric analysis on a CytoFLEX instrument (Beckman Coulter). For each group, *n* = 3–4 mice were used.

### Measurement of cellular ATP levels

Cellular ATP levels were quantified using a luciferase‐based ATP Assay Kit (Beyotime, Shanghai, China). Cell lysates were cleared by centrifugation, and the resulting supernatants were reacted with the substrate solution. Luminescence was recorded on a luminometer with an integration time of 10 s per well. ATP concentrations were interpolated from a standard curve of known ATP standards and normalized to total protein content to correct for cell number variability. For each group, *n* = 4–6 mice were used.

### Statistical analysis

All statistical analyses were performed using graphpad prism software (version 8.0) (GraphPad Software, San Diego, CA, USA). For comparisons between two groups of normally distributed data, a two‐tailed Student's *t*‐test was applied. For comparisons among three groups, one‐way ANOVA followed by Tukey's *post hoc* test was used. The Mann–Whitney *U*‐test was used for data that did not follow a normal distribution. Data are presented as mean ± SEM. Statistical significance was defined as *P < 0.05, **P < 0.01, and ***P < 0.001. All experiments were performed with one or two independent biological replicates, and the exact number of mice per group (*n*) is specified for each assay as detailed above.

## Results

### Re‐analysis of scRNA‐seq data reveals the transcriptional profiles of neonatal M‐MDSCs following BCG vaccination

**Fig. 1:**
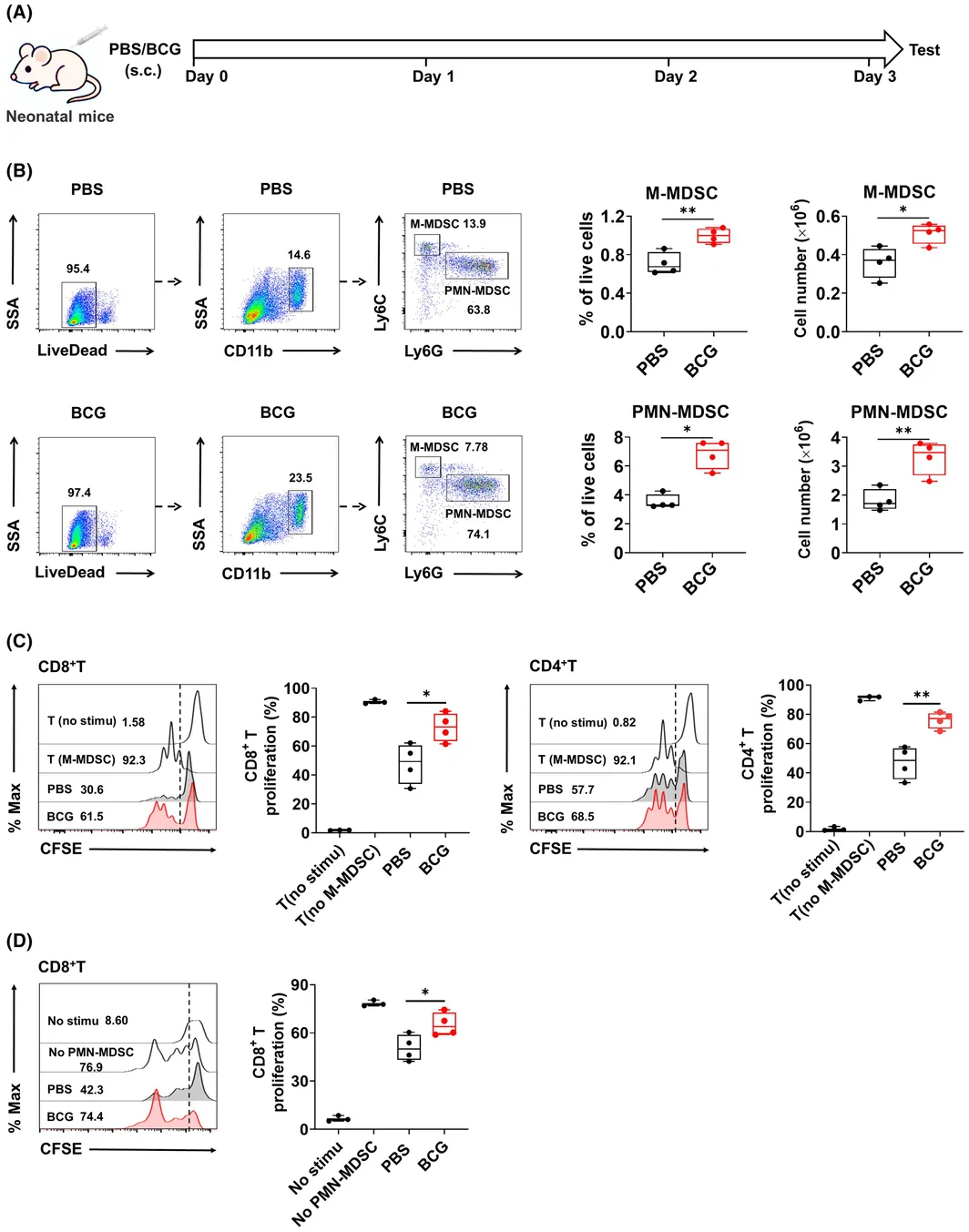
BCG abrogates the immunosuppressive function of M‐MDSCs and PMN‐MDSCs. C57BL/6 neonates received a single subcutaneous injection of BCG (5 × 10^5^ CFU) or PBS (control) at 3–4 days of age and were euthanized 3 days post‐vaccination for splenic analysis. (A) Experimental timeline. (B) Representative flow cytometry plots (left) and the frequency/absolute number (right) of M‐MDSCs and PMN‐MDSCs (*n* = 4 mice per group from two independent experiments; each dot represents one mouse). (C) CD3/CD28‐induced proliferation of CD8^+^ and CD4^+^ T cells co‐cultured with M‐MDSCs. Controls included unstimulated T cells and stimulated T cells without M‐MDSCs. Proliferation was assessed by CFSE dilution; representative plots (left) and quantification (right) are shown (*n* = 3–4 mice per group from two independent experiments). (D) Antigen‐specific proliferation of CD8^+^ T cells co‐cultured with PMN‐MDSCs, with controls as in (C). CFSE‐based proliferation results are presented as representative plots (left) and summary statistics (right) (*n* = 3–4 mice per group from two independent experiments). For the box‐and‐whisker plots in (B–D), the box represents the 25th–75th percentile, the line inside the box the median, and the whiskers the minimum to maximum values. *P < 0.05, **P < 0.01, using a two‐tailed Student's *t*‐test or Mann–Whitney *U*‐test. BCG, Bacillus Calmette‐Guérin; CFSE, carboxyfluorescein succinimidyl ester; M‐MDSC, monocytic myeloid‐derived suppressor cell; PBS, phosphate‐buffered saline; PMN‐MDSC, polymorphonuclear myeloid‐derived suppressor cell.

We previously reported that BCG impairs the immunosuppressive function of both M‐MDSCs and PMN‐MDSCs in neonates through an mTOR‐HIF1α‐dependent upregulation of glycolysis [16, 17]. Consistent with these findings, neonatal mice (postnatal days 3–4) received a single subcutaneous injection of 5 × 10^5^ CFU BCG (Fig. 1A). BCG vaccination significantly expanded splenic M‐MDSCs and PMN‐MDSCs (Fig. 1B) and, as expected, attenuated their capacity to suppress T‐cell proliferation (Fig. 1C,D).

**Fig. 2:**
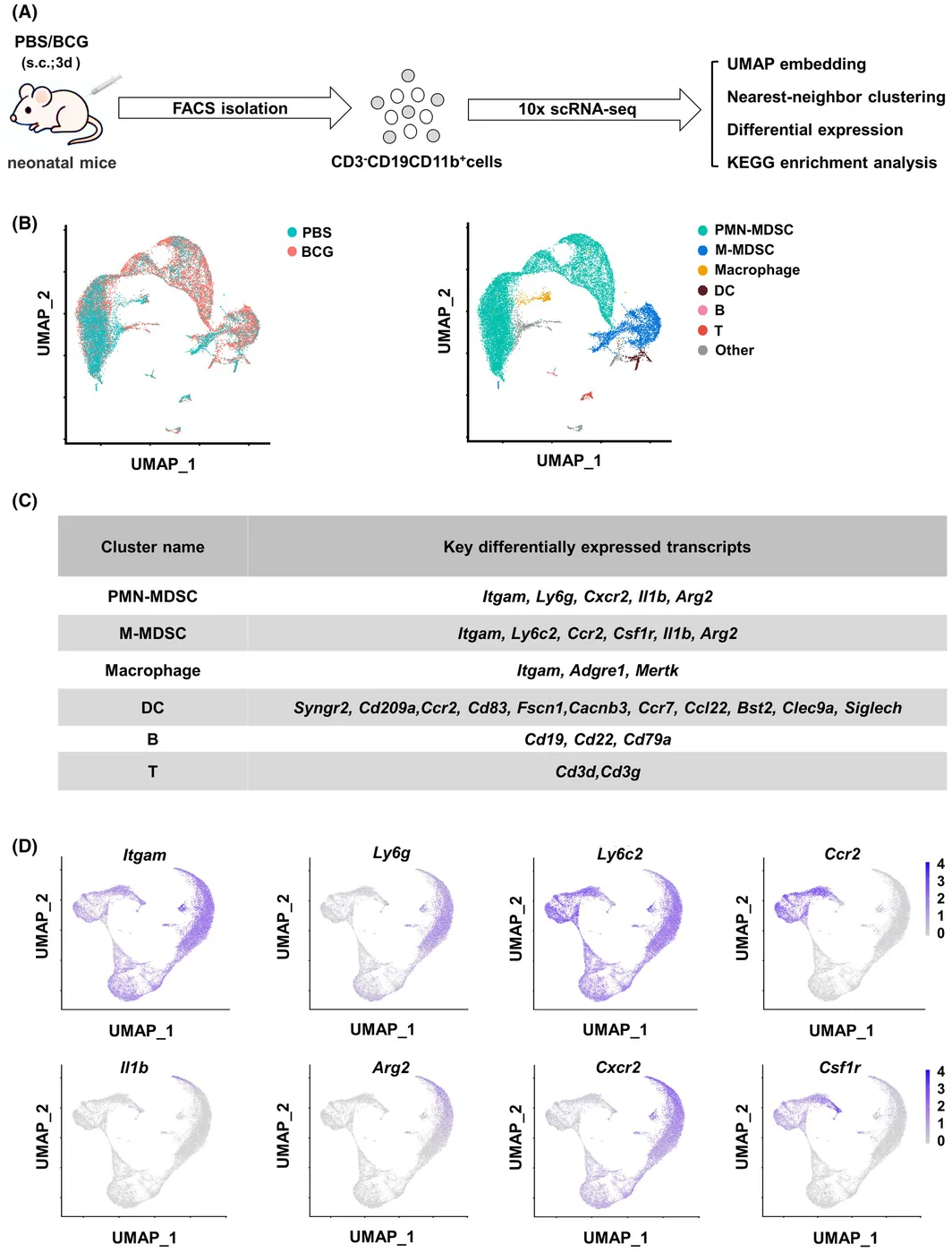
Major cell populations identified from scRNA‐seq re‐analysis using marker gene expression. (A) Schematic overview of the scRNA‐seq experimental design and analytical workflow. (B) UMAP visualization of CD3^−^CD19^−^CD11b^+^ splenic myeloid cells from PBS‐treated and BCG‐vaccinated mice. Cells are jointly embedded but colored separately by experimental condition. (C) Key marker genes used for cell‐type annotation. (D) UMAP projection of MDSCs, showing scaled expression levels of selected signature genes across identified cell types. Color intensity corresponds to the mean normalized expression for each gene. Data are from one biological replicate with no technical replicates. BCG, Bacillus Calmette–Guérin; DC, dendritic cells; FACS, fluorescence‐activated cell sorting; MDSC, myeloid‐derived suppressor cell; PBS, phosphate‐buffered saline; scRNA‐seq, single‐cell RNA sequencing; UMAP, uniform manifold approximation and projection.

**Fig. 3:**
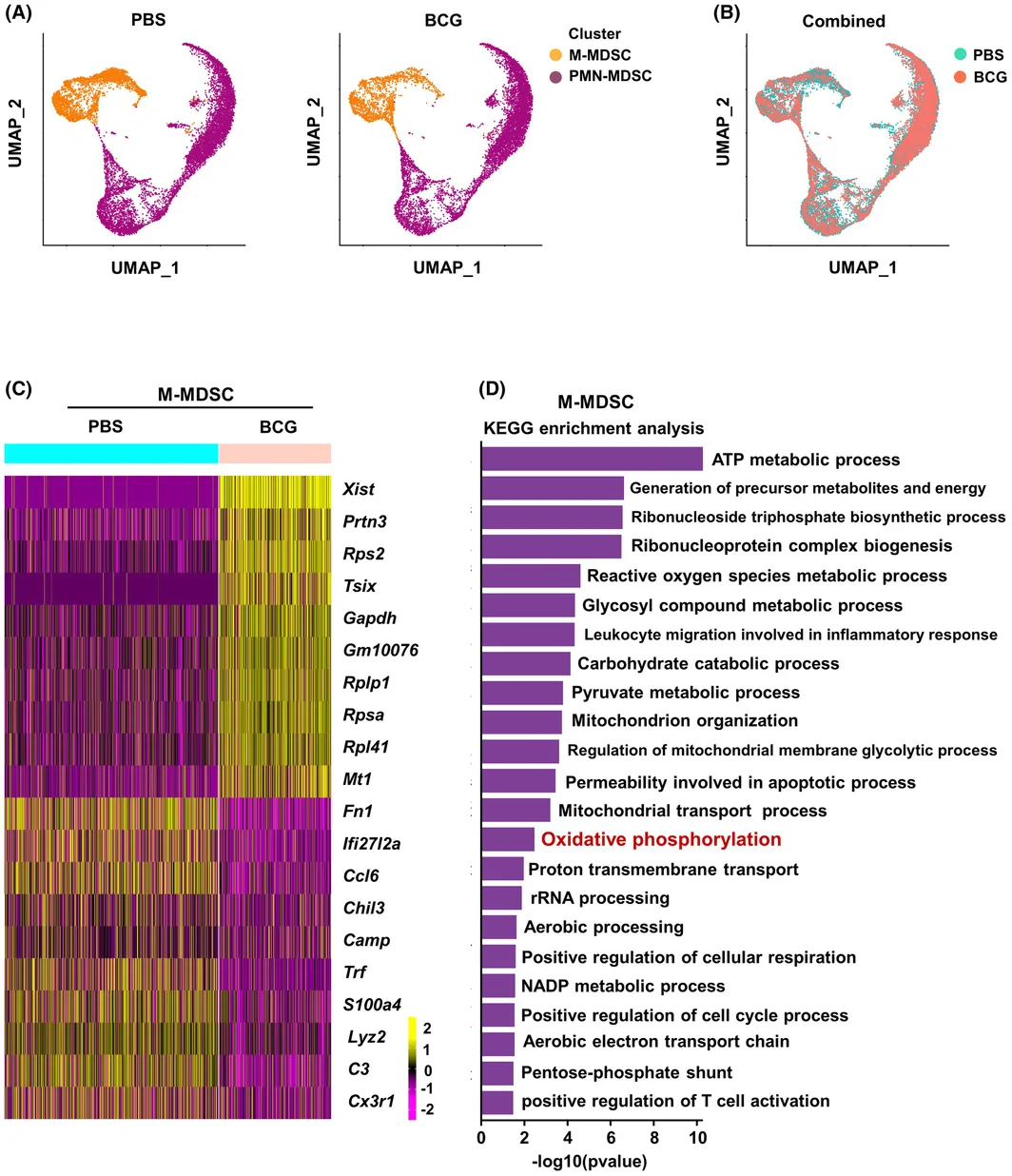
Re‐analysis of scRNA‐seq data reveals the transcriptional profiles of neonatal M‐MDSCs following BCG vaccination. (A, B) UMAP projections of splenic MDSCs from PBS‐treated and BCG‐vaccinated neonates. All CD3^−^CD19^−^CD11b^+^ cells are embedded together; each condition is shown separately. Colors indicate cell types. (C) Heatmap of row‐scaled expression of the most significantly altered genes in M‐MDSCs from PBS‐ or BCG‐vaccinated neonates. (D) Enrichment scores of KEGG metabolic pathways in M‐MDSCs from BCG‐vaccinated versus PBS‐control mice. Data are from one pooled scRNA‐seq experiment with no technical replicates. BCG, Bacillus Calmette‐Guérin; KEGG, Kyoto Encyclopedia of Genes and Genomes; MDSC, myeloid‐derived suppressor cell; M‐MDSC, monocytic myeloid‐derived suppressor cell; PBS, phosphate‐buffered saline; scRNA‐seq, single‐cell RNA sequencing; UMAP, uniform manifold approximation and projection.

To explore whether BCG affects neonatal MDSCs via additional pathways, we re‐analyzed our previously published scRNA‐seq dataset (GSE234153), which profiled splenic CD3^−^CD19^−^CD11b^+^ myeloid cells from BCG‐vaccinated and PBS‐treated control neonates (Fig. 2A) [16]. Using canonical correlation analysis (CCA), we identified major cell populations based on marker gene expression (Fig. 2B–D). Unsupervised clustering resolved two distinct MDSC subsets, PMN‐MDSCs and M‐MDSCs, according to their transcriptional signatures (Figs 3A,B and 2C,D). PMN‐MDSCs showed high expression of *Itgam, Ly6g, Cxcr2, Il1b*, and *Arg2*; M‐MDSCs prominently expressed *Itgam, Ly6c2, Ccr2, Csf1r, Il1b*, and *Arg2* (Fig. 2C,D). We then focused on the M‐MDSC subset. A heatmap of the most significantly altered genes revealed clear transcriptional differences between PBS‐ and BCG‐derived M‐MDSCs (Fig. 3C). KEGG pathway analysis identified oxidative phosphorylation (OXPHOS) as a novel significantly enriched pathway in M‐MDSCs after BCG vaccination (Fig. 3D). Collectively, these data provide a high‐resolution transcriptomic landscape of neonatal M‐MDSCs and highlight a BCG‐induced metabolic rewiring that includes OXPHOS as a previously unappreciated component.

### BCG‐induced transcriptional reprogramming of neonatal PMN‐MDSCs identified from scRNA‐seq re‐analysis

**Fig. 4:**
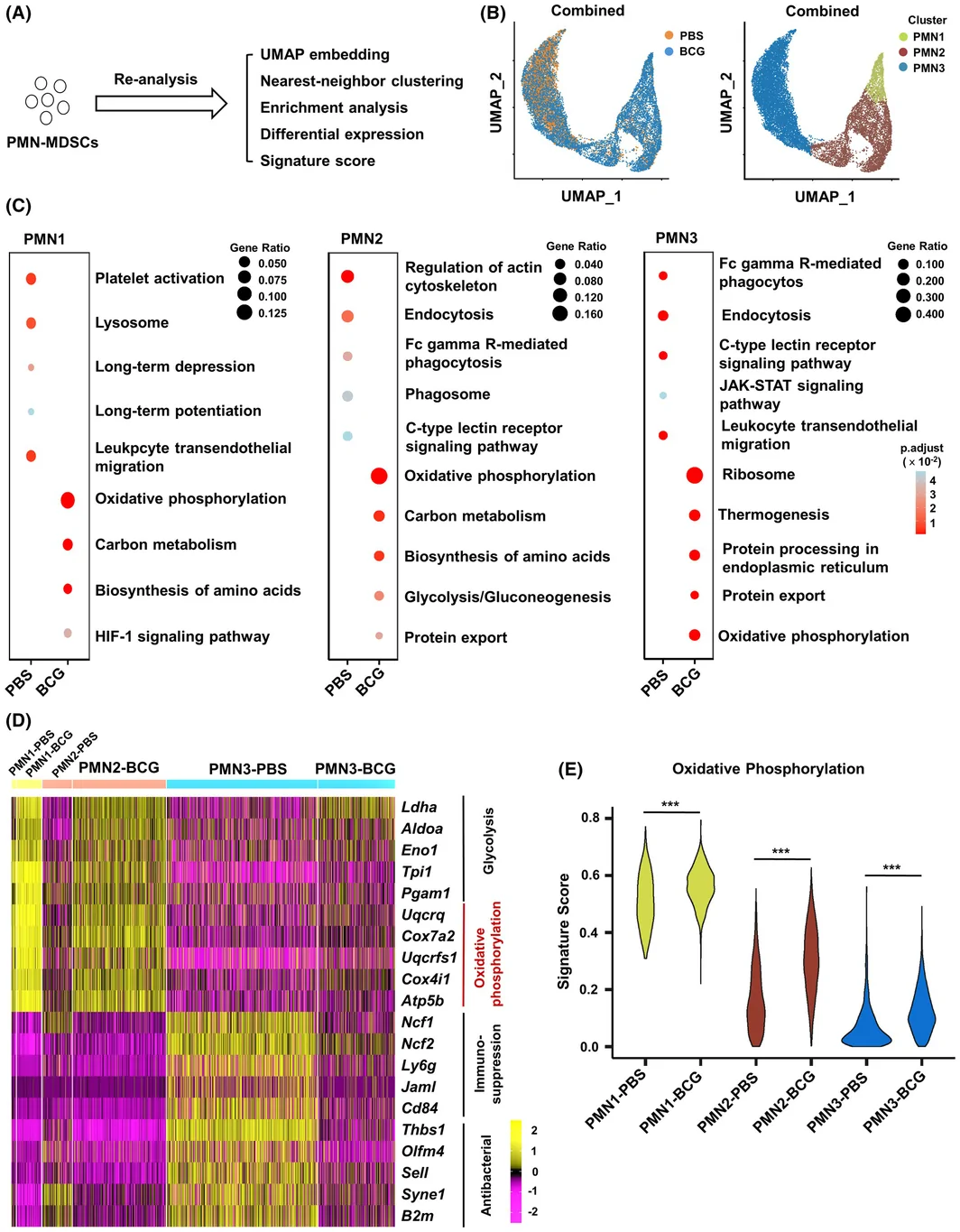
BCG‐induced transcriptional reprogramming of neonatal PMN‐MDSCs identified from scRNA‐seq re‐analysis. (A) Schematic overview of the experimental design. (B) UMAP projections of PMN‐MDSCs from PBS‐treated and BCG‐vaccinated neonates (same embedding, separate plots). Colors indicate subclusters (PMN1, PMN2, PMN3). (C) Gene Ontology (GO) analysis of differentially expressed genes in PMN‐MDSC clusters from BCG‐vaccinated versus PBS‐derived cells. (D) Heatmap of selected OXPHOS‐related genes in PMN‐MDSCs from PBS‐treated and BCG‐vaccinated groups. (E) OXPHOS activity scores for each PMN‐MDSC cluster. Comparisons are between PBS‐treated and BCG‐vaccinated mice. Data are from one pooled scRNA‐seq experiment with no technical replicates. In (E), *P*‐values were generated using a two‐tailed Student's *t*‐test. Significance is indicated as *P* < 0.001 (***). BCG, Bacillus Calmette‐Guérin; GO, Gene Ontology; OXPHOS, oxidative phosphorylation; PBS, phosphate‐buffered saline; PMN‐MDSC, polymorphonuclear myeloid‐derived suppressor cell; scRNA‐seq, single‐cell RNA sequencing; UMAP, uniform manifold approximation and projection.

Because PMN‐MDSCs are the predominant MDSC subset in neonates, we next re‐examined these cells. Unsupervised clustering of the scRNA‐seq data defined three transcriptionally distinct PMN‐MDSC subpopulations, PMN1, PMN2, and PMN3, based on their enriched gene signatures (Fig. 4A,B). Gene Ontology (GO) analysis showed that OXPHOS was one of the most significantly enriched pathways across all PMN clusters after BCG vaccination (Fig. 4C). Accordingly, OXPHOS‐related genes were markedly upregulated in PMN1 and PMN2 subsets from BCG‐vaccinated mice relative to PBS controls (Fig. 4D). Metabolic scoring further indicated that PMN1 had the highest baseline OXPHOS activity, and BCG vaccination significantly increased OXPHOS scores in all three clusters (Fig. 4E). Together, these re‐analyzed data indicate that BCG vaccination remodels the transcriptional landscape and promotes OXPHOS in neonatal PMN‐MDSCs.

### Enhanced OXPHOS underlies the loss of immunosuppressive function in neonatal BCG‐derived M‐MDSCs

**Fig. 5:**
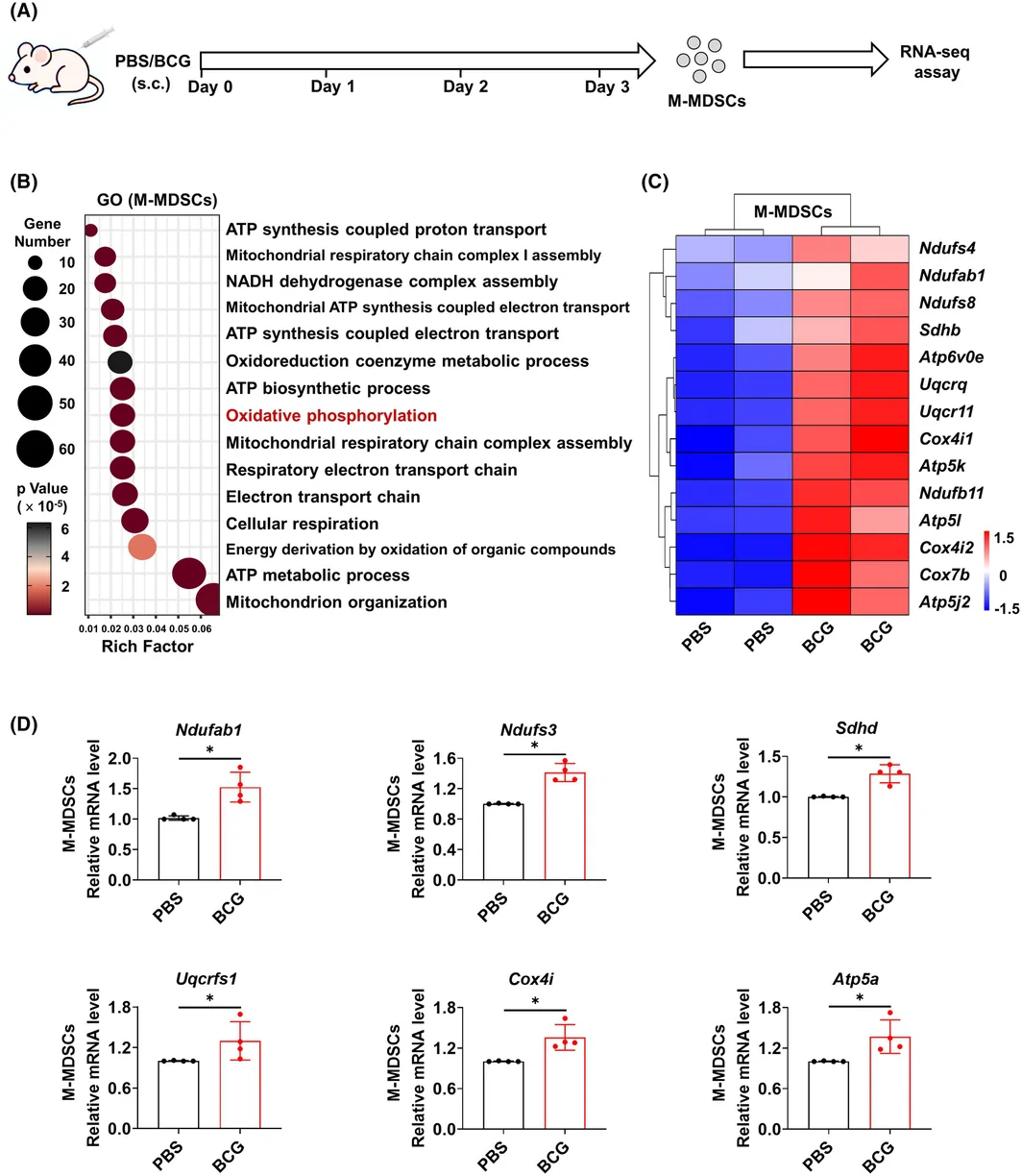
Re‐analysis of RNA‐seq data reveals that BCG treatment enhances OXPHOS in neonatal M‐MDSCs. (A) Experimental timeline. (B) GO analysis of differentially expressed genes in M‐MDSCs from BCG‐vaccinated versus PBS‐treated neonates. (C) Heatmap of OXPHOS‐related gene expression in M‐MDSCs on day 3 post‐BCG (log_2_FC > 1, adj.*P* < 0.05). (D) qRT‐PCR validation of OXPHOS gene expression (*n* = 4 mice per group from two independent experiments; each point represents one mouse). Data are mean ± SEM. *P < 0.05 using Mann–Whitney *U* test. BCG, Bacillus Calmette‐Guérin; GO, Gene Ontology; M‐MDSC, monocytic myeloid‐derived suppressor cell; OXPHOS, oxidative phosphorylation; PBS, phosphate‐buffered saline; qRT‐PCR, quantitative real‐time polymerase chain reaction; RNA‐seq, RNA sequencing; SEM, standard error of the mean.

To further delineate the molecular mechanisms of BCG‐induced metabolic reprogramming in M‐MDSCs, we re‐analyzed our previously datasets of bulk RNA‐seq on splenic M‐MDSCs (GSE330508) isolated from BCG‐vaccinated and PBS‐treated neonatal mice (Fig. 5A) [17]. GO analysis revealed significant enrichment of OXPHOS‐related pathways (Fig. 5B). Consistent with this, key OXPHOS genes encoding electron transport chain components, including *Ndufab1* (complex I), *Uqcr11* (complex III), *Cox4i1* (complex IV), *Atp5k*, and *Atp5l* (complex V), were markedly upregulated in BCG‐exposed M‐MDSCs compared with PBS controls (Fig. 5C). qRT‐PCR confirmed increased expression of *Ndufab1, Sdhd, Uqcrfs1*, and *Cox4i* (Fig. 5D).

**Fig. 6:**
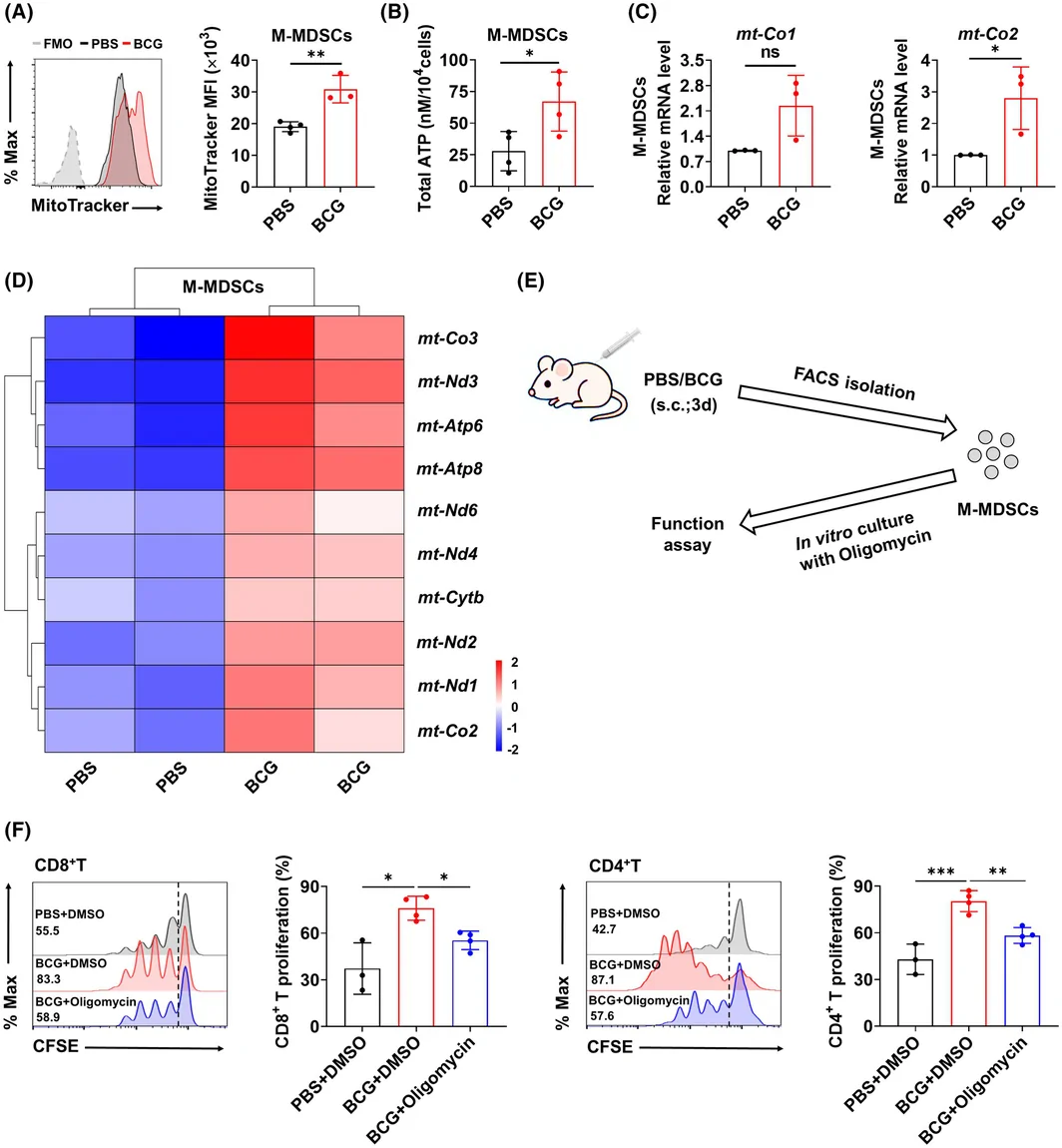
Enhanced OXPHOS underlies the loss of immunosuppressive function in BCG‐treated neonatal M‐MDSCs. (A) MitoTracker Red staining on day 1 post‐ BCG vaccination. Representative histograms and mean fluorescence intensity (MFI) summary (*n* = 3–4 mice per group from two independent experiments). (B) Total ATP levels in M‐MDSCs on day 3 (*n* = 4 mice per group from two independent experiments). (C) qRT‐PCR validation of mitochondrial gene expression (*n* = 3 mice per group from one independent experiments). (D) Heatmap of mitochondrial gene expression in M‐MDSCs on day 3 post‐BCG (log_2_FC > 1, adj.*P* < 0.05). (E) Schematic of the *in vitro* OXPHOS inhibition assay. (F) Suppressive capacity of M‐MDSCs from PBS, BCG, or oligomycin‐treated conditions on CD8^+^ and CD4^+^ T‐cell proliferation (*n* = 3–4 mice per group from two independent experiments). Data are mean ± SEM. *P < 0.05, **P < 0.01, ***P < 0.001 and ns, not significant using a two‐tailed Student's *t*‐test, Mann–Whitney *U*‐test or one‐way ANOVA followed by Tukey's *post hoc* test. ANOVA, analysis of variance; ATP, adenosine triphosphate; BCG, Bacillus Calmette‐Guérin; MFI, mean fluorescence intensity; M‐MDSC, monocytic myeloid‐derived suppressor cell; OXPHOS, oxidative phosphorylation; PBS, phosphate‐buffered saline; qRT‐PCR, quantitative real‐time polymerase chain reaction; SEM, standard error of the mean.

Mitochondrial membrane potential, assessed by MitoTracker staining, was significantly higher in BCG‐vaccinated M‐MDSCs (Fig. 6A), indicating mitochondrial hyperpolarization. Total cellular ATP levels were also elevated (Fig. 6B). To determine whether this enhanced bioenergetic state was accompanied by increased mitochondrial gene transcription, we performed qRT‐PCR for mitochondrial‐encoded genes. BCG‐vaccinated M‐MDSCs displayed higher mRNA levels of *mt‐Co1* and *mt‐Co2*, which encode subunits of complex IV, relative to controls (Fig. 6C). Bulk RNA‐seq further confirmed that additional mitochondrial genes encoding electron transport chain components, including *mt‐Nd1, mt‐Nd2, mt‐Nd3, mt‐Nd4*, *mt‐Nd6* (complex I), *mt‐Cytb* (complex III), *mt‐Co2*, *mt‐Co3* (complex IV), *mt‐Atp6*, and *mt‐Atp8* (complex V), were substantially upregulated in BCG‐exposed M‐MDSCs compared with PBS controls (Fig. 6D). Together, these results indicate that BCG vaccination promotes enhanced oxidative metabolism in neonatal M‐MDSCs.

To test whether increased OXPHOS directly impairs immunosuppressive capacity, we treated BCG‐exposed M‐MDSCs with the OXPHOS inhibitor oligomycin (Fig. 6E). Oligomycin partially restored their ability to suppress both CD8^+^ and CD4^+^ T‐cell proliferation (Fig. 6F). Thus, BCG‐induced augmentation of OXPHOS impairs the immunosuppressive activity of neonatal M‐MDSCs.

### Enhanced OXPHOS mediates the diminished immunosuppressive function of neonatal PMN‐MDSCs following BCG treatment

**Fig. 7:**
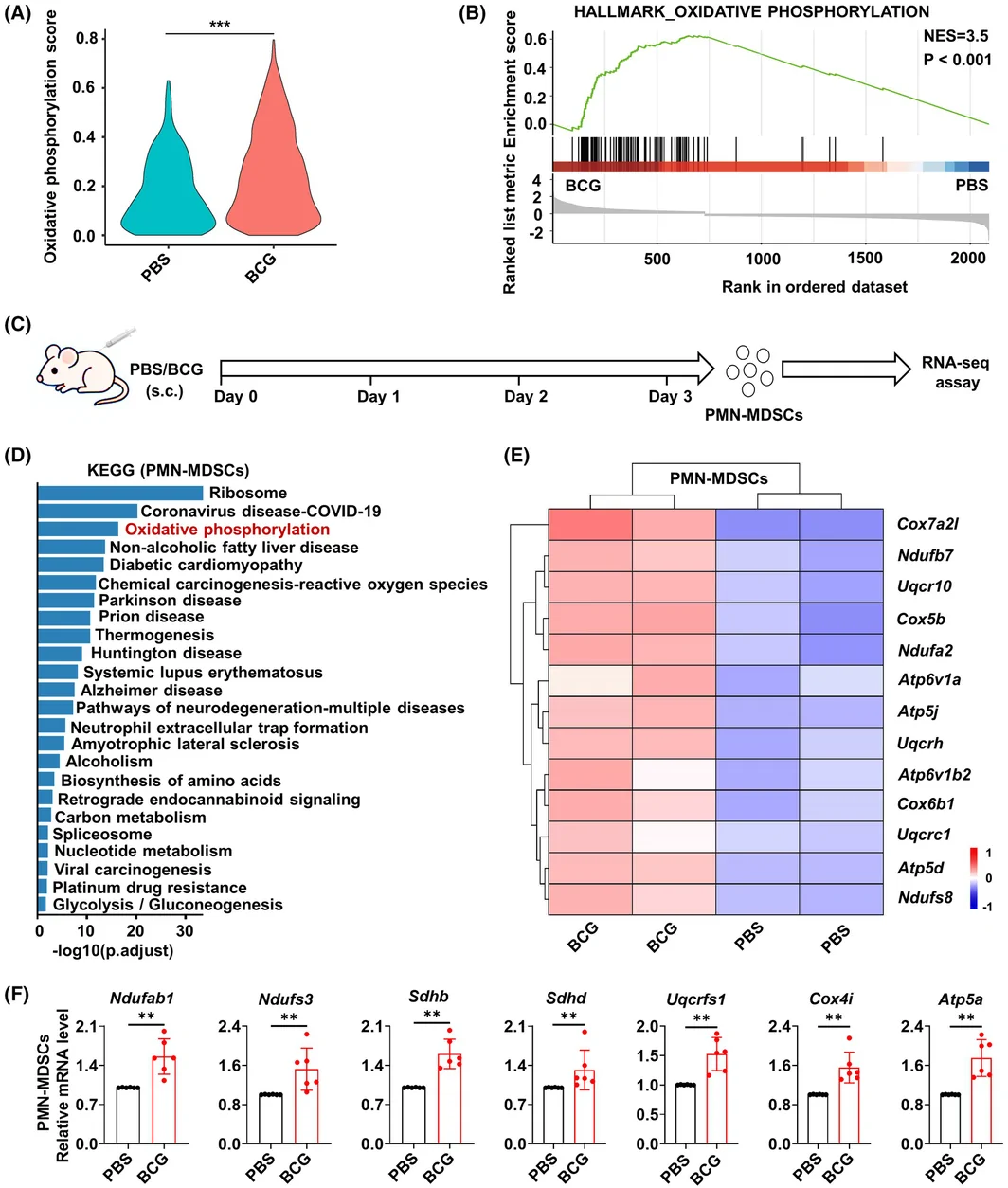
Re‐analysis of RNA‐seq data reveals that BCG treatment upregulates OXPHOS in neonatal PMN‐MDSCs. (A) Violin plot comparing OXPHOS activity scores in PMN‐MDSCs between groups, derived from scRNA‐seq data. (B) Gene set enrichment analysis (GSEA) plot depicting enrichment of the OXPHOS pathway in PMN‐MDSCs. (C) Experimental timeline. (D) KEGG pathway analysis of RNA‐seq data from PMN‐MDSCs. (E) Heatmap of OXPHOS gene expression in PMN‐MDSCs from PBS‐ and BCG‐treated mice (RNA‐seq). (F) qRT‐PCR validation of OXPHOS gene expression (*n* = 6 mice per group from two independent experiments). Data are mean ± SEM. **P < 0.01 and ***P < 0.001 using a two‐tailed Student's *t*‐test or Mann–Whitney *U*‐test. BCG, Bacillus Calmette‐Guérin; GSEA, gene set enrichment analysis; KEGG, Kyoto Encyclopedia of Genes and Genomes; OXPHOS, oxidative phosphorylation; PBS, phosphate‐buffered saline; PMN‐MDSC, polymorphonuclear myeloid‐derived suppressor cell; qRT‐PCR, quantitative real‐time polymerase chain reaction; RNA‐seq, RNA sequencing; scRNA‐seq, single‐cell RNA sequencing; SEM, standard error of the mean.

PMN‐MDSCs from BCG‐vaccinated mice showed significantly higher OXPHOS activity than PBS controls, as quantified by scRNA‐seq (Fig. 7A); this was confirmed by GSEA (Fig. 7B). We then re‐analyzed our previously datasets of bulk RNA‐seq isolated PMN‐MDSCs (GSE234153) from control and BCG‐treated neonates (Fig. 7C). KEGG pathway re‐analysis of the bulk RNA‐seq identified OXPHOS as one of the most significantly enriched pathways in PMN‐MDSCs after BCG vaccination (Fig. 7D). Key OXPHOS transcripts, *Cox7a2l, Cox5b, Uqcrh*, and *Atp5d*, were upregulated (Fig. 7E), and qRT‐PCR confirmed increased expression of *Ndufab1, Sdhb, Uqcrfs1*, and *Cox4i* (Fig. 7F).

**Fig. 8:**
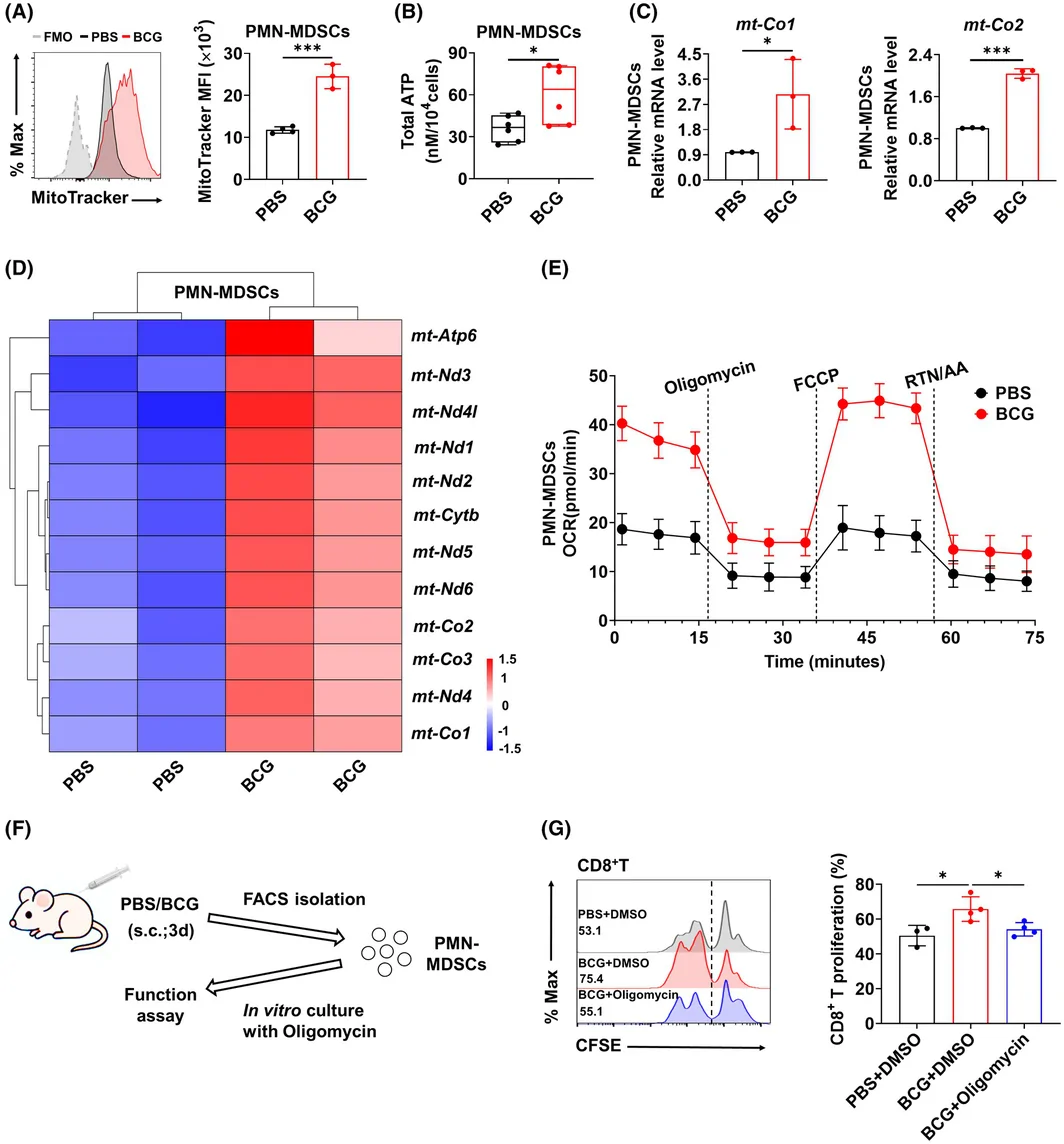
Enhanced OXPHOS mediates the diminished immunosuppressive function of neonatal PMN‐MDSCs following BCG treatment. (A) MitoTracker staining on day 1 post‐vaccination. Representative histograms and MFI summary (*n* = 3–4 mice per group from two independent experiments). (B) Total ATP levels on day 3 (*n* = 6 mice per group from two independent experiments). (C) qRT‐PCR validation of mitochondrial gene expression (*n* = 3 mice per group from two independent experiments). (D) Heatmap of mitochondrial gene expression in PMN‐MDSCs on day 1 post‐BCG (log_2_FC > 1, adj.*P* < 0.05). (E) OCR measured by Seahorse analysis on day 3 (*n* = 4 mice per group from two independent experiments). (F) Schematic of the *in vitro* OXPHOS inhibition assay. (G) Antigen‐specific CD8^+^ T‐cell proliferation co‐cultured with PMN‐MDSCs from PBS, BCG, or oligomycin‐treated conditions (*n* = 3–4 mice per group from two independent experiments). For the box‐and‐whisker plots in (B), the box represents the 25th–75th percentile, the line inside the box the median, and the whiskers the minimum to maximum values. *P < 0.05 and ***P < 0.001, using a two‐tailed Student's *t*‐test, Mann–Whitney *U*‐test or one‐way ANOVA followed by Tukey's *post hoc* test. ANOVA, analysis of variance; ATP, adenosine triphosphate; BCG, Bacillus Calmette‐Guérin; OCR, oxygen consumption rate; OXPHOS, oxidative phosphorylation; PBS, phosphate‐buffered saline; PMN‐MDSC, polymorphonuclear myeloid‐derived suppressor cell; qRT‐PCR, quantitative real‐time polymerase chain reaction.

Mitochondrial membrane potential, assessed by MitoTracker staining, was also elevated in BCG‐vaccinated PMN‐MDSCs (Fig. 8A), accompanied by an increase in total cellular ATP levels (Fig. 8B). qRT‐PCR analysis revealed significantly enhanced expression of the mitochondrial‐encoded genes *mt‐Co1* and *mt‐Co2* in BCG‐exposed PMN‐MDSCs (Fig. 8C). This transcriptional upregulation was further corroborated by bulk RNA‐seq, which showed marked increases in multiple mitochondrial genes encoding electron transport chain subunits including *mt‐Nd1 ‐ mt‐Nd6* (complex I), *mt‐Cytb* (complex III), *mt‐Co1 ‐ mt‐Co3* (complex IV), and *mt‐Atp6* (complex V) (Fig. 8D). Seahorse metabolic flux analysis revealed a substantial increase in oxygen consumption rate (OCR) in PMN‐MDSCs from BCG‐vaccinated neonates (Fig. 8E), providing functional evidence of enhanced mitochondrial respiration. Collectively, these metabolic and transcriptional readouts point to a coordinated elevation of oxidative metabolism in neonatal PMN‐MDSCs following BCG vaccination.

To establish a causal relationship between elevated OXPHOS and loss of immunosuppressive function, we treated BCG‐exposed PMN‐MDSCs with oligomycin (Fig. 8F). Oligomycin partially restored their capacity to suppress CD8^+^ T‐cell proliferation (Fig. 8G). Collectively, these data demonstrate that BCG‐augmented OXPHOS compromises the immunosuppressive capacity of neonatal PMN‐MDSCs.

## Discussion

This work establishes OXPHOS as a previously unappreciated metabolic axis through which BCG vaccination reprograms neonatal MDSCs. BCG upregulated electron transport chain genes (*Ndufab1, Sdhd, Uqcrfs1, Cox4i*) and mitochondrial‐encoded transcripts in both M‐MDSC and PMN‐MDSC subsets. In parallel, it increased mitochondrial membrane potential, ATP levels, and oxygen consumption rates. Pharmacological inhibition of OXPHOS with oligomycin partially restored the immunosuppressive function of BCG‐exposed MDSCs, indicating a causal link between BCG‐driven metabolic changes and functional modulation. Collectively, these findings offer a mechanistic basis for the heterologous immunomodulatory actions of BCG vaccination in early life.

Importantly, this work largely builds upon re‐analyses of our previously scRNA‐seq and bulk RNA‐seq (GSE234153, GSE330508) datasets [16, 17], complemented by new experimental validations including qRT‐PCR, MitoTracker staining, ATP assays, and Seahorse analysis on PMN‐MDSCs. The transcriptomic data for both MDSC subsets were derived from our prior studies; the novelty of the current study therefore lies not in the generation of new sequencing data, but in a deliberate shift of analytical focus from glycolysis to OXPHOS, a pathway not previously examined in depth. Through targeted pathway analysis combined with pharmacological inhibition, we demonstrate that OXPHOS upregulation is not merely correlated with, but functionally causal for, the loss of MDSC immunosuppressive capacity. Thus, although the raw sequencing data overlap with our earlier publications, the biological question, analytical strategy, and mechanistic validation presented here are distinct and substantially extend our previous work.

The concurrent use of bulk and single‐cell RNA‐seq was motivated by two methodological considerations. First, bulk RNA‐seq offers greater statistical power to validate OXPHOS signatures at the population level, as scRNA‐seq can underestimate low‐abundance transcripts [33]. Second, it allowed us to confirm that the OXPHOS enrichment observed in specific subclusters was not an artifact of subcluster heterogeneity but was also evident across the bulk population [34]. Beyond validation, bulk RNA‐seq provided quantitative log_2_(fold change) and adjusted *P*‐values for individual genes, enabling more stringent statistical assessment and independently confirmed the OXPHOS pathway enrichment via KEGG and GO analyses. Importantly, the bulk results consistently recapitulated the scRNA‐seq findings, showing OXPHOS gene upregulation in both MDSC subsets across platforms. This cross‐platform concordance supports the conclusion that the OXPHOS signature is a bona fide biological feature of BCG‐exposed MDSCs, not a technical artifact of single‐cell analysis. Of note, our previous observation of glycolysis gene upregulation was also confirmed in these re‐analyzed datasets [16, 17], indicating that BCG simultaneously drives both glycolytic and oxidative metabolic programs in neonatal MDSCs.

It is important to clarify that our experimental model, analyzing MDSCs at a single early time point (day 3 post‐vaccination), does not conform to the classical definition of trained immunity, which typically requires a resting phase followed by restimulation with an unrelated pathogen [5]. We therefore do not claim that our data directly demonstrate trained immunity. Instead, we interpret the early metabolic rewiring, including OXPHOS, as an acute response that may precede and potentially prime long‐term epigenetic and functional adaptations. Any connection to trained immunity remains speculative at this stage and is raised only as a possible parallel, not as an established mechanism in our system.

Our results place OXPHOS at the center of BCG‐induced MDSC modulation. They extend our earlier work showing that BCG upregulates glycolysis in neonatal MDSCs via an mTOR‐HIF1α‐dependent pathway [16, 17]. The concurrent activation of both glycolysis and OXPHOS suggests a broad increase in metabolic activity that may drive MDSCs from an immunosuppressive, immature state toward a more activated phenotype. This parallel report that BCG‐induced trained immunity in monocytes also involves both glycolysis and OXPHOS [5], implying a conserved metabolic feature of innate immune memory across cell types. Ehlers et al. recently highlighted that OXPHOS dominates the metabolic profile of neonatal myeloid cells [35]; our data show that BCG amplifies this pre‐existing trait, thereby reducing MDSC suppressive capacity.

The translational implications of these findings, however, must be interpreted with caution. Although BCG vaccination has been linked to reduced all‐cause mortality in low‐birth‐weight infants [4], the present data derive entirely from a mouse model, and extrapolation to human neonates, particularly preterm infants, would be premature without further validation. To date, human neonatal MDSCs, especially from preterm populations, remain insufficiently accessible to confirm the OXPHOS‐MDSC axis in humans. Nonetheless, identifying OXPHOS as a metabolically modifiable node in MDSC function provides a rationale for future translational exploration. Emerging single‐cell technologies that require minimal blood volumes [10] offer a feasible path toward testing our observations in human samples; until such evidence emerges, any clinical interpretation must remain preliminary.

Several limitations should be noted. First, MitoTracker Red FM is potential‐sensitive and does not directly measure mitochondrial mass. We therefore attribute the increased fluorescence in BCG‐vaccinated MDSCs to mitochondrial hyperpolarization rather than an absolute increase in organelle content. To support this interpretation, we obtained three independent lines of orthogonal evidence: upregulation of mitochondrial‐encoded genes (*mt‐Nd1–Nd6, mt‐Cytb, mt‐Co1–Co3, mt‐Atp6/Atp8*) in both MDSC subsets, as confirmed by qRT‐PCR and bulk RNA‐seq; elevated total cellular ATP levels; and a direct increase in oxygen consumption rate in PMN‐MDSCs measured by Seahorse analysis. These convergent data collectively point to enhanced oxidative metabolism, independent of dye‐related artifacts. While mtDNA copy number or TOM20/VDAC quantification could further distinguish mass from potential in future work, we consider the current multi‐layered evidence sufficient to support our main conclusions. Second, the upstream signals linking BCG recognition to mitochondrial biogenesis and OXPHOS enhancement are not fully defined. While our previous work implicated the mTOR‐HIF1α pathway in BCG‐induced glycolysis, OXPHOS augmentation may involve distinct or overlapping pathways. Third, because of the scarcity of neonatal M‐MDSCs, we could not perform Seahorse analysis on this subset and relied on indirect metabolic measurements. Fourth, although our pharmacological inhibition experiments establish causality in mouse cells, the clinical relevance of the BCG‐OXPHOS‐MDSC axis remains speculative without access to sufficient human neonatal MDSCs, particularly from preterm infants. New single‐cell approaches that require very small blood volumes offer a promising route for translating our findings to human studies [10].

## Conclusion

In summary, this work establishes OXPHOS as a previously underappreciated pathway through which BCG reprograms neonatal MDSCs. By driving a metabolic shift toward oxidative metabolism, BCG vaccination attenuates the immunosuppressive function of these cells, with dual implications for both beneficial heterologous protection and potential inflammatory risks. A more comprehensive understanding of this immunometabolism crosstalk may guide the design of safer, more effective vaccination strategies for vulnerable neonatal populations, particularly preterm infants, who face the highest burden of both infectious and inflammatory diseases.

## Conflict of interest

The authors declare no conflict of interest.

## Author contributions

YC conceived and designed the project; HL acquired, curated, and formally analyzed the data; YC and HL interpreted the data; HL wrote the original draft; YC supervised the study, provided resources and funding, and reviewed and edited the manuscript. All authors contributed to the revision and approved the final version.

## Data Availability

The raw scRNA‐seq and bulk RNA‐seq data supporting the findings of this study are deposited in the Gene Expression Omnibus (GEO) under accession numbers GSE234153 and GSE330508. All other data that support the findings are available from the corresponding author upon reasonable request.
